# Characteristics of sexually experienced HIV testers aged 18 to 32 in rural South Africa: baseline results from a community-based trial, NIMH Project Accept (HPTN 043)

**DOI:** 10.1186/1471-2458-14-1164

**Published:** 2014-11-13

**Authors:** Lucia Knight, Nuala McGrath, Heidi van Rooyen, Hilton Humphries, Alastair van Heerden, Linda Richter

**Affiliations:** School of Public Health, University of the Western Cape, P Bag X17, Bellville, 7535 South Africa; Academic Unit of Primary Care and Population Sciences, and Department of Social Statistics and Demography, Mailpoint 805, Southampton General Hospital, Tremona Road, Southampton, SO16 6YD UK; Africa Centre for Health and Population Studies, University of KwaZulu-Natal, PO Box 198, Mtubatuba, KZN 3935 South Africa; HIV/AIDS STIs and TB Programme, Human Sciences Research Council, P.O Box 90, Msunduzi, 3200 South Africa; Developmental Pathways of Health Research Unit, Department of Paediatrics, School of Clinical Medicine, Faculty of Health Sciences, University of the Witwatersrand, Johannesburg, South Africa; HIV/AIDS STIs and TB Programme, Human Sciences Research Council, 750 Francois Road, Cato Manor, Durban, 4001 South Africa; DST-NRF Centre of Excellence in Human Development, University of the Witwatersrand, Johannesburg, South Africa

**Keywords:** HIV testing, Rural, South Africa, Young people

## Abstract

**Background:**

Young people in South Africa are at high risk of HIV infection and yet may have more limited access to prevention and treatment services than others in the population. Testing facilitates the sharing of prevention messages but also enables the linkage to care and treatment of those who test positive and therefore has wider public health implications.

**Methods:**

This baseline survey conducted in 2005 for a community randomized trial in rural KwaZulu-Natal explored factors associated with a history of ever, repeat and recent testing amongst sexually debuted men and women aged 18 to 32 years.

**Results:**

Over 35% of this rural population ever tested for HIV, with men less likely to ever (unadjusted OR 0.26, 95% CI: 0.21-0.32) and repeatedly test than women (adjusted OR (aOR) 0.68, 95% CI: 0.48-0.97). Men aged 24–28 years (aOR 2.02, 95% CI: 1.10-3.71) and 29–32 years (aOR 2.69, 95% CI: 1.46-4.94) were more likely to ever test than those <20 years. Those who reported having discussed HIV with others had significantly greater odds of reporting ever (men’s aOR 2.83, 95% CI: 1.63-4.89; women’s aOR 3.36, 95% CI: 2.50-4.53), recent (irrespective of sex, aOR 2.87, 95% CI: 2.02-4.09) and repeat testing (aOR 2.02, 95% CI: 1.28-3.19).

**Conclusion:**

These findings highlight the need for novel youth- and men-friendly testing services and emphasises the importance of discussions about HIV in the home and community to encourage testing.

## Background

South Africa has amongst the highest national adult HIV prevalence rate in the world, 12.3% in 2012 [1]. National survey data show that peak prevalence shifted in women from 25 to 29 years in 2008 to 30 to 35 years in 2012, possibly relating to increased life-expectancy resulting from antiretroviral treatment programmes [1]. Nonetheless, the burden of known new infections is largest amongst young people, particularly women under the age of 25 [2, 3].

Increasing access to and uptake of HIV testing among the general population, and especially young people, is a critical step in managing HIV and AIDS in South Africa. HIV testing has implications for prevention by encouraging risk reduction [4, 5] and for linkage to care [6]. Therefore the focus is increasingly on early and repeat testing, prior to symptomatic illness, either in voluntary or provider initiated counselling and testing (PICT) contexts. Despite a large national HIV testing campaign (2010–2011) and universal access to free testing through public health facilities, surveys show that fewer than 50% of adults know their status, with more than twice as many women reporting testing than men [1]. Prior to the campaign, younger age groups (15 to 25 years) [7–9] were least likely to have tested and fewer linked to medical care [10].

Studies in South Africa show evidence of high risk behaviours: two out of three of those who refuse testing reported HIV risk behaviour, including multiple sexual partners, exchanging sex for money, injecting drug usage and having had a sexually transmitted infections [11]. Despite the increased risk of HIV infection, perceptions of low risk exist amongst the general population [12] and young people [13, 14]. This low risk perception is coupled with psychological barriers to testing, such as fear of the result, stigma and a perception that testing was for the symptomatic [8]. These and service-related barriers such as lack of confidentiality and distance to and delays at testing centres [15] account for low testing rates in South Africa. These barriers along with negative staff attitudes and inconvenient clinic hours, identified as key barriers to facility use by adolescents suggest an urgent need to address access to testing [16].

This paper provides insight into the HIV testing behaviour of young people aged 18 to 32 years prior to the implementation of a community-based trial. Few studies report on this broader age range, which reflects peak risk groups, such as women over 25, in South Africa [7, 17]. Further, the uptake of HIV testing in rural communities is poorly reported on, with a greater focus on factors associated with HIV testing in urban and peri-urban contexts [11, 18, 19]. This study addresses these gaps by assessing the impact of various factors on ever, recent and repeat testing amongst 18–32 year old men and women in a high HIV prevalence, rural South African context.

## Methods

This paper uses data from the baseline individual-level survey from the NIMH Project ACCEPT (HPTN 043) community randomized trial conducted in rural Vulindlela, KwaZulu-Natal (KZN) in 2005, similar analyses from the urban site have been published elsewhere [19]. This paper builds on the data published previously for the urban sites by providing a detailed analysis of the factors associated with testing in the rural South African context, and in a setting characterised by higher HIV prevelance. The HIV prevention trial included rural and urban communities in South Africa and communities in Tanzania, Zimbabwe and Thailand that were randomized to receive either a community-based HIV counselling and testing (HCT) intervention plus standard clinic-based HCT, or standard HCT alone. The intervention had three major strategies: (1) to make VCT more available in community settings (reduce barriers to testing; (2) to engage the community through outreach; and (3) to provide post-test support (reduce perceived adverse consequences of testing).

A random sample of households was selected from eight study communities, selected from within Vulindlela, with a combined population of approximately 40 000 rural residents. In each selected household, one resident respondent, 18 to 32 years was randomly selected and enrolled. The total sample size was 2596.

All respondents provided informed consent and ethical clearance for the Project Accept study was provided by the University of California at Los Angeles South General Institutional Review Board and the University of the Witwatersrand.

To explore testing history we created three binary outcome variables: the first compared those who self-reported ever having an HIV test against those who do not report testing, the second those who self-reported having multiple tests against those who report ever testing once, and finally those self-reporting testing in the last six months, or recently, to those reporting ever testing. The baseline survey included systematically collected data about the reasons for testing for all those who ever tested.

The characteristics of the sample are described in Table 1. Recent behavioural characteristics indicative of increasing risk of HIV in the South African context were included to determine associations with recent testing, these included lower age at first sex, increased number of recent and lifetime sexual partners and no or inconsistent condom use. Indirect risk factors included in the analysis were self-reported drug and alcohol use which are sometimes associated with increased risk of HIV acquisition. Data on injecting drug use was collected but negligible and therefore excluded from the analysis and data about sexually transmitted infections was not collected. Variables were included that reflected individuals’ attitudes to or perception of HIV. The first of these was a binary indicator for whether or not people report ever having had a discussion about HIV. This question was phrased in terms of a conversation with someone about HIV/AIDS. The respondent was also provided with a number of possible topics the conversation may have included including prevention, risk, testing and HIV and the community. This question was asked both in terms of lifetime discussions and discussions in the last 6 months. The question is limited in that it does not provide us with information about the timing or nature of the discussion. Secondly, two categorical variables were created based on summary means scores; one involved the six items used to measure social norms relating to HIV and the other comprised 22 items created to measure stigma associated with HIV [20]. Each was created using three categories: unfavourable, intermediate and favourable for social norms and low, intermediate or high levels of stigma towards HIV. The final variable was created using a score from questions about perceptions and knowledge of ART, this variable was also categorical and included those who had not heard about ART and then three different levels (low, medium or high) of positive perception about ART.

**Table 1:** **Sample characteristics (N = 2255) of sexually debuted 18–32 year olds**

Characteristic			Male % (N = 923)	Female % (N = 1332)	p-values
**Testing**	**Ever testing**	Yes	18	46	<0.001*
		No	82	54	
	**Recent testing**	Yes	7	17	0.41
		No	93	83	
	**Repeat testing**	Yes	8	26	<0.001*
		No	92	74	
**Demographics**	**Age**	18-20 years	31	25	<0.001*
		21-24 years	29	26	
		25-28 years	20	24	
		29-32 years	20	26	
	**Household or individual Socio-economic status**	Low	37	37	0.47
		Medium	49	51	
		High	14	12	
	**Years of education**	< 7 years	8	10	0.02*
		8-10 years	30	25	
		11-12 years	57	60	
		Tertiary	5	4	
	**Occupation**	Employed	39	24	<0.001*
		Unemployed	32	59	
		Student	29	17	
	**Income from work**	Yes	60	43	<0.001*
		No	40	57	
	**Children under care**	0	67	24	<0.001*
		1	15	31	
		2+	18	45	
	**Cohabiting**	Living apart	25	18	<0.001*
		No partner	70	67	
		Cohabiting	6	15	
**Behaviour indicative of increased risk for HIV & perceptions of HIV**	**No. of lifetime partners**	1 partner	10	34	<0.001*
		2 partners	14	30	
		3-4 partners	25	28	
		5+ partners	51	8	
	**Age at first sex**	< 15 years	31	14	<0.001*
		16-17 years	37	36	
		18 + years	32	50	
	**Lifetime alcohol**	Yes	80	29	<0.001*
		No	20	71	
	**Lifetime drugs**	Yes	30	2	<0.001*
		No	70	98	
	**Recent condom use**	No partner	26	22	<0.001*
		Never	26	37	
		Sometimes	20	17	
		Always	28	25	
	**Ever discussion about HIV**	No	21	24	0.11
		Yes	79	76	
	**Social norms for testing**	Unfavourable	24	33	<0.001*
		Intermediate	42	38	
		Favourable	34	29	
	**Stigma score**	Low	20	35	<0.001*
		Intermediate	37	34	
		High	42	30	
	**Perception of ART**	Not heard of ART	32	27	0.003*
		Low	38	27	
		Medium	21	20	
		High	19	26	

*Significant at the 95% confidence level.

The analyses considered only baseline respondents reporting ever having sex and therefore at risk of acquiring HIV. The three outcome variables were tested for univariate association with socio-demographic and behavioural characteristics associated with increased risk of HIV and perceptions of HIV. In order to better elucidate sex-specific characteristics associated with HIV testing, we present analyses of ever testing stratified by gender. For the multivariate analyses, age and gender (where appropriate) was included in all models *a priori*. Recent and repeat testing models were not split by gender because of the very small numbers reporting recent testing overall and men reporting repeat testing. All other variables were removed one at a time if inclusion did not significantly improve the fit of the model. Additional analyses present self-reported data about the most commonly selected reasons for choosing to have an HIV test (from a predefined list). These results provide insight into both the reason for testing. Analyses were conducted using STATA (STATACORP, version 11.0, College Station, TX) software.

## Results

The analysis for this paper is based on 2,255 (89% of total sample) young respondents who reported ever having sex. The majority of these sexually active respondents were women (59%), reflecting the same proportion of women in the entire sample.

Table 1 shows that 35% of respondents reported ever having tested for HIV (n = 789), significantly more women reported ever (46%) and repeat (26%) testing than men (18%, p = <0.001 and 8%, p = <0.001 respectively). Of those who tested, 62% had last tested more than 6 months ago and 38% more recently. In terms of repeat testing, 53% had tested more than once and 14% reported being HIV-positive. Men were significantly more likely to report behaviours that may be indicative of increased risk of HIV than women. Over 75% of men reported more than 3 lifetime sexual partners versus 34% for women. In addition men reported earlier sexual debut than women. Conversely, men seem to report more effective condom use in the six months prior to the interview than women, more men report not having a partner and always using a condom while fewer report never and only sometimes using condoms.

Of those reporting ever testing 84% gave one reason for testing, only 11% and one percent reported two or three reasons respectively. Table 2 shows the foremost reason for HIV testing was a desire to know one’s status and the second was non-voluntary testing. Non-voluntary testing was self-defined but likely included PICT, antenatal routine testing or insurance testing. Neither testing because of ill health or sexual partner’s risk were associated with gender. However, more women than men reported testing because of having children, non-voluntary testing and wanting to know their HIV status (for all p < 0.01; 100%, 98% and 74% of the total reporting these reasons were women).

**Table 2:** **Ranking of most commonly cited reasons for choosing to have an HIV test (N = 805)** ^**1**^

	Total %	Female %	N	p-value ^a^
**Wanted to know status**	51	74	408	0.001*
**Non-voluntary**	19	98	149	<0.001*
**Having children**	17	100	137	<0.001*
**Sick**	12	75	95	0.31
**Sexual partner had risky behaviour**	0.03	89	26	0.19

^a^Chi-square tests for comparison of men and women.

*Significant at the 95% confidence level.

^1^The questions for Table 2 allow for multiple responses so the N may overlap as discussed in the text. The total N is 805.

### HIV testing among men

Of the 923 sexually active male participants, only 170 (18%) reported ever testing for HIV. A significant association exists between ever testing and age; men over 25 years have increased odds of ever testing for HIV (Table 3). In the adjusted model, there was no significant association between ever reporting HIV testing and education, socio-economic status or number of children under care. There was an association between testing and men’s occupation with male students having reduced odds of testing. Fewer than 4% (n = 3) were tested at their workplace and therefore this is unlikely to have influenced this finding.

**Table 3:** **Socio-demographic factors, recent indicators of behavioural risk and perceptions of HIV associated with reporting ever testing amongst men and women**

			Men N = 923				Women N = 1332			
		Characteristic	Test % (n = 170)	Unadjusted OR (95% CI)	Adjusted OR* (95% CI)	LRT p value	Test % (n = 619)	Unadjusted OR (95% CI)	Adjusted OR** (95% CI)	LRT p value
**Demographics**	**Age**	18-20 years	15	1	1	0.01	27	1	1	<0.001
		21-24 years	29	**2.21 (1.33-3.69)**	1.67 (0.96-2.93)		29	1.07 (0.79-1.45)	0.86 (0.62-1.21)	
		25-28 years	25	**2.95 (1.74-5.00)**	**2.02 (1.10-3.71)**		22	0.75 (0.55-1.02)	**0.55 (0.39-0.69)**	
		29-32 years	31	**4.02 (2.40-6.73)**	**2.69 (1.46-4.94)**		23	**0.67 (0.49-0.91)**	**0.48 (0.33-0.69)**	
	**Socio-economic status**	Low	34	0.85 (0.59-1.22)			38	1.13 (0.89-1.42)		
		Medium	51	1			50	1		
		High	15	1.04 (0.63-1.71)			11	0.91 (0.64-1.28)		
	**Years of education**	< 7 years	5	0.54 (0.26-1.13)			11	1.26 (0.88-1.82)		
		8-10 years	24	**0.67 (0.45-0.99)**			27	1.24 (0.96-1.60)		
		11-12 years	62	1			57	1		
		Tertiary	8	1.78 (0.91-3.46)			5	1.35 (0.79-2.31)		
	**Occupation**	Employed	38	1	1	0.06	21	1	1	<0.001
		Unemployed	48	0.94 (0.65-1.36)	1.03 (0.71-1.51)		63	**1.45 (1.12-1.89)**	1.26 (0.96-1.66)	
		Student	14	**0.31 (0.19-0.51)**	**0.53 (0.29-0.96)**		15	1.02 (0.72-1.44)	0.77 (0.51-1.16)	
	**Income from work**	Yes	66	1			41	1		
		No	34	**0.70 (0.49-0.98)**			59	1.15 (0.92-1.43)		
	**Children under care**	0	62	1			18	1	1	<0.001
		1	20	1.50 (0.97-2.33)			34	**1.06 (1.52-2.78)**	**2.11 (1.55-2.88)**	
		2+	18	1.08 (0.69-1.69)			48	**1.83 (1.38-2.43)**	**2.00 (1.49-2.69)**	
	**Cohabiting**	Living apart	75	1			67	1		
		No partner	18	**0.63 (0.41-0.97)**			17	0.86 (0.65-1.15)		
		Cohabiting	6	1.11 (0.55-2.22)			16	1.18 (0.87-1.61)		
**Behaviour indicative of increased risk for HIV & Perceptions of HIV**	**No. of lifetime partners**	1 partner	8	0.57 (0.31-1.07)			35	0.82 (0.53-1.26)		
		2 partners	39	**0.36 (0.20-0.67)**			28	0.70 (0.45-1.08)		
		3-4 partners	20	**0.57 (0.37-0.87)**			28	0.80 (0.51-1.24)		
		5 + partners	65	1			9	1		
	**Age at first sex**	< 15 years	33	0.97 (0.64-1.45)			16	1.35 (0.97-1.88)		
		16-17 years	32	0.76 (0.51-1.15)			36	1.02 (0.81-1.29)		
		18 < years	35	1			49	1		
	**Lifetime alcohol**	Yes	76	1			31	1		
		No	24	1.34 (0.90-1.99)			69	0.85 (0.67-1.08)		
	**Lifetime drugs**	Yes	31	1.02 (0.71-1.47)			2	1.63 (0.72-3.69)		
		No	69	1			98	1		
	**Ever discussion about HIV**	No	10	1	1	<0.001	87	1	1	<0.001
		Yes	90	**2.83 (1.67-4.80)**	**2.83 (1.63-4.89)**		13	**3.34 (2.53-4.42)**	**3.36 (2.50-4.53)**	
	**Social norms for testing**	Unfavourable	27	1.40 (0.90-2.19)			31	0.93 (0.70-1.22)		
		Intermediate	44	1.32 (0.89-1.96)			40	1.14 (0.87-1.48)		
		Favourable	29	1			29	1		
	**Stigma Score**	Low	22	1.22 (0.78-1.90)			34	0.87 (0.67-1.14)		
		Intermediate	38	1.08 (0.74-1.57)			34	0.91 (0.69-1.19)		
		High	40	1			32	1		
	**Perception of ART**	Not heard of ART	21	1			22	1	1	<0.001
		Low	36	**2.20 (1.40-3.46)**			28	**1.43 (1.06-1.92)**	1.18 (0.86-1.63)	
		Medium	22	**1.68 (1.02-2.76)**			19	1.28 (0.93-1.77)	1.00 (0.71-1.41)	
		High	21	**1.75 (1.05-2.90)**			31	**2.03 (1.50-2.74)**	**1.63 (1.17-2.26)**	

*Model is adjusted for Age, Occupation, and Report of a discussion about HIV.

**Model is adjusted for Age, Occupation, Children under care, Report of a discussion about HIV and Perception of ART.

Bold indicates Significant at the 95% confidence level.

No risk behaviour variables were associated with ever testing in men in the adjusted model. Neither were stigma, social norms or perceptions of ART. Discussions about HIV were significantly associated with ever testing, men who had had discussions were almost three times more likely to have ever tested (aOR 2.83, 95% CI: 1.63-4.89) compared to those men who had not discussed HIV.

### HIV testing among women

Of the 1,332 sexually active women in the study, 619 (46%) reported ever testing for HIV. Age was associated with reporting ever testing, women over 25 years had lower odds of testing (Table 3). Occupation was significantly associated with testing among women in a univariable model but was not significant in a multivariable model adjusted for age and those factors associated in the univariable. Women with children under their care had higher odds of ever testing than those with no children in the adjusted model.

Like men, no behavioural risk factors nor stigma or social norms were associated with ever testing among women. Women reporting a discussion about HIV were more than three times more likely to have ever tested compared with those who did not have a discussion (aOR 3.36, 95% CI: 2.50-4.53). Women reporting a high perception of ART had higher odds of reporting HIV testing (aOR 1.63, 95% CI: 1.17-2.26) relative to women who had not heard of ART.

### Recent and repeat testing

Table 4 shows the factors associated with recent and repeat testing in both an unadjusted and adjusted model. The adjusted model shows that unlike ever testing, no difference exists by gender for recent testing, although women were more likely to repeat test. Those older than 25 had lower odds of recent testing than other age groups, but no association exists between age and repeat testing. Employed people had lower odds of recent testing than the unemployed. In contrast socio-economic status is associated with repeat testing only, those with low and high socio-economic status have higher odds of repeat testing than those in the middle category.

**Table 4:** **Socio-demographic factors, recent indicators of behavioural risk and perceptions of HIV associated with recent (last 6 months) and repeated testing for HIV amongst those ever testing (N = 789)**

Characteristic			Recent Test % (n = 296)	Unadjusted OR (95% CI)	Adjusted OR* (95% CI)	LRT p value	Repeated % (n = 417)	Unadjusted OR (95% CI)	Adjusted OR** (95% CI)	LRT p value
**Demographics**	**Gender**	Male	21	0.94 (0.66-1.34)	1.19 (0.81-1.74)	<0.001	18	**0.68 (0.48-0.95)**	**0.68 (0.48-0.97)**	0.02
		Female	79	1	1		82	1	1	
	**Age**	18-20 years	29	1	1	0.10	26	1	1	
		21-24 years	30	0.79 (0.53-1.16)	0.77 (0.51-1.18)		28	0.82 (0.56-1.21)	0.89 (0.60-1.34)	
		25-28 years	20	**0.59 (0.38-0.89)**	**0.61 (0.38-0.98)**		23	0.85 (0.56-1.28)	0.81 (0.52-1.24)	
		29-32 years	21	**0.60 (0.39-0.90)**	**0.61 (0.38-0.97)**		24	0.80 (0.54-1.20)	0.76 (0.50-1.16)	
	**Socio-economic status**	Low	37	0.99 (0.72-1.35)			41	**1.51 (1.11-2.05)**	**1.49 (1.10-2.02)**	0.01
		Medium	50	1			46	1	1	
		High	13	1.07 (0.67-1.69)			13	1.57 (0.999-2.47)	**1.61 (1.02-2.54)**	
	**Years of education**	< 7 years	11	1.32 (0.81-2.15)			12	1.51 (0.92-2.48)		
		8-10 years	29	1.23 (0.88-1.72)			25	0.93 (0.67-1.29)		
		11-12 years	56	1			58	1		
		Tertiary	4	0.59 (0.29-1.19)			6	1.10 (0.59-2.04)		
	**Occupation**	Employed	19	**0.49 (0.34-0.70)**	**0.50 (0.34-0.73)**	<0.001	27	1		
		Unemployed	65	1	1		57	1.02 (0.74-1.41)		
		Student	17	1.03 (0.68-1.55)	0.86 (0.55-1.34)		16	1.21 (0.80-1.82)		
	**Income from work**	Yes	41	**0.68 (0.51-0.91)**			48	1		
		No	59	1			52	0.89 (0.67-1.18)		
	**Children under care**	0	31	1.31 (0.92-1.87)			24	**0.69 (0.49-0.98)**		
		1	29	0.95 (0.67-1.35)			31	0.88 (0.63-1.23)		
		2+	38	1			44	1		
	**Cohabiting**	No partner	18	1.10 (0.74-1.62)			18	1.06 (0.72-1.54)		
		Living apart	66	1			69	1		
		Cohabiting	16	1.27 (0.84-1.92)			13	0.81 (0.54-1.22)		
**Behaviour indicative of increased risk for HIV & Perceptions of HIV**	**Recent sexual partners**	0 Partners	21	1.07 (0.75-1.53)			21	1.09 (0.76-1.54)		
		1 Partner	72	1			72	1		
		2 + Partners	7	0.93 (0.53-1.62)			7	0.80 (0.47-1.38)		
	**Recent condom use**	No partners	21	0.93 (0.61-1.42)			21	0.80 (0.53-1.21)	0.80 (0.52-1.23)	0.04
		Never	29	0.76 (0.52-1.12)			30	**0.63 (0.43-0.91)**	**0.62 (0.42-0.91)**	
		Sometimes	22	0.99 (0.64-1.49)			18	**0.58 (0.39-0.88)**	**0.57 (0.37-0.88)**	
		Always	28	1			30	1		
	**No. of lifetime partners**	1 partner					32	1.33 (0.90-1.96)		
		2 partners					23	1		
		3-4 partners					25	0.96 (0.65-1.42)		
		5 + partners					20	1.07 (0.70-1.63)		
	**Age at first sex**	< 15 years					20	1.11 (0.76-1.63)		
		16-17 years					35	1.05 (0.77-1.44)		
		18 < years					45	1		
	**Lifetime alcohol**	Yes	42	1.11 (0.83-1.48)			39	0.86 (0.64-1.13)		
		No	58	1			61	1		
	**Lifetime drugs**	Yes	7	0.76 (0.44-1.30)			8	0.88 (0.53-1.46)		
		No	93	1			92	1		
	**Ever discussion about HIV**	No	19	1	1	<0.001	91	1	1	<0.001
		Yes	81	**2.62 (1.86-3.69)**	**2.87 (2.02-4.09)**		9	**2.06 (1.34-3.18)**	**2.02 (1.28-3.19)**	
	**Social norms for testing**	Unfavourable	27	1			31	1		
		Intermediate	43	1.28 (0.90-1.81)			40	0.88 (0.63-1.23)		
		Favourable	29	1.21 (0.83-1.77)			29	1.00 (0.69-1.44)		
	**Stigma Score**	Low	34	1			33	1		
		Intermediate	33	0.82 (0.57-1.16)			36	1.01 (0.72-1.43)		
		High	33	0.86 (0.60-1.22)			31	0.80 (0.57-1.14)		
	**Perception of ART**	Not heard of ART	25	1.07 (0.72-1.60)			20	**0.50 (0.33-0.74)**	**0.51 (0.33-0.79)**	<0.001
		Low	24	**0.64 (0.43-0.94)**			26	**0.49 (0.34-0.72)**	**0.49 (0.33-0.75)**	
		Medium	20	0.86 (0.57-1.31)			20	**0.60 (0.40-0.91)**	**0.59 (0.38-0.91)**	
		High	31	1			35	1	1	

*Model is adjusted for Gender, Age, Occupation and Report of a discussion about HIV.

**Model is adjusted for Gender, Age, Socio-economic status, Recent condom use, Report of a discussion about HIV and Perception of ART.

Bold indicates Significant at the 95% confidence level.

Unlike ever testing among women, that showed a relationship between having children and ever testing, no other socio-demographic variables significantly affected the final models for recent or repeat testing.

While no risk factors were associated with ever or recent testing, frequency of condom use was associated with repeat testing. Those reporting no recent sexual partners or no or infrequent recent condom use had lower odds of repeat testing for HIV compared to those always using condoms.

Neither social norms nor stigma were associated with recent or repeat testing. In contrast, those with no knowledge or a low or medium positive perception of ART all had lower odds of repeat testing than those with high positive perception. Similar to findings for ever testing, those who report a discussion about HIV had significantly greater odds of both recent and repeat testing compared to those who reported no discussions.

## Discussion

In this representative baseline sample of sexually active 18-32-year-olds from rural KwaZulu-Natal, 35% of respondents report ever testing for HIV in 2005, with gender, age and discussions about HIV emerging as important factors in determining HIV testing.

Studies show poor uptake of testing in rural populations in South Africa [9, 21]. This is particularly concerning in high HIV prevalence contexts such as KZN [21]. Despite using the same methodology and a similar sample, HIV testing in this rural community is lower than in the Soweto (urban) Project Accept site (48%) over the same period [19]. Alternative models of testing are therefore required to ensure the maximum testing coverage in rural populations.

Gendered patterns of testing demonstrate that men had lower rates of ever and repeat testing than women. Similar gender differential were observed in a 2003 national survey of youth 15 to 24 years, although overall rates of testing are lower than observed here, possibly because of lower testing rates in men under 24 [17]. Gendered patterns of testing highlight the need to increase testing opportunities for men, especially younger men.

Motivations for testing are also gendered with significantly more women reporting non-voluntary and antenatal testing as opportunities to test. This may explain the reduced rate of testing in women without children. Evidence from the urban site and others demonstrates that, in addition to PICT in pregnancy, women’s motivations may be linked to the perceived benefits for their infant [19, 22–24]. It is essential to give *all* women the opportunity to know their status and address their risks for HIV [17].

The gender imbalance in both testing and motivations is compounded by evidence from sub-Saharan Africa that women have more contact with health facilities than men, mainly through reproductive and child health services [25, 26]. Moreover, health services are not considered male-friendly spaces, with operating hours and provider attitudes that may lack sensitivity to men’s needs [27]. Consequently, fewer men than women are establishing and disclosing their HIV status [28], acknowledging their symptoms, and accessing treatment across Africa [29–31].

Lower testing rates among younger men in this high prevalence context are a concern. Despite lower rates of HIV than among women of a similar age [1], men reported higher rates of risk behaviours than women, making them an important target group for testing, risk awareness and prevention messaging. Young men experience similar barriers to health service access as older men [8, 32, 33], highlighting the need for more acceptable and accessible services for all men [19, 34].

The association between conversations about HIV and testing is noted in both national and urban South African studies of young people [17, 19]. A joint analysis of the South African Project Accept data [35] shows findings similar to those observed here with discussions associated with a significantly increased odds of testing (between 1.29 to 3 times). To account for the influence of reverse causality, an analysis including both recent testing and discussions observes that the relationship still exists. While the nature of these discussions or with whom they took place is hard to discern, evidence suggests that discussions about HIV play a role in the decision to test, possibly through normalising HIV and reducing the fear associated with testing.

The observed association between a positive perception of ART and ever testing in women and repeat testing is corroborated by qualitative data from the same period and communities showing that access to ART influences the decision to test for HIV [36]. Again, causality is not implied because ART access may follow testing. However, the association is also supported by evidence from a rural KZN cohort where access to ART increased over time (2005 to 2011) alongside knowledge of HIV status [37].

Although, no relationship was observed between stigma and testing, the relative importance of access to ART coupled with the influence of discussions on testing has the potential to shift risk perceptions and stigma by encouraging disclosure and conversations about HIV amongst individuals, couples, families and communities.

Except for a relationship between repeat testing and recent condom use evidence for associations between risk and testing do not exist in this and the urban data [19]. The observed association suggests a reduction in risk behaviours because of repeat testing or that cautious people test more often. A rural HIV survey of young people 15–24 years (2006 to 2011) demonstrated a relationship between testing and reduced HIV incidence likely due to behaviour change and the potential positive impact of testing in this context [38].

While there was minimal evidence for association between socio-economic variables and testing, employed people did show a lower odds of recent testing than the unemployed, this is possibly linked to access to testing which is traditionally provided in public facilities during work hours and not always easy for those with employment to access. This issue of access may also be related to the observed relationship between socio-economic status and repeat testing that suggests that those with low and high socio-economic status have higher odds of repeat testing, possibly because of access to free public testing by those with low status and the ability of those with higher status to afford alternatives to free public testing. This highlights the need to provide alternatives to standard opportunities for testing everyone for free.

### Limitations

The data from this paper was collected in 2005 and it is likely that the situation has changed. At the time, PICT was not policy and ART availability was not widespread. Certainly this and other research shows that increased access to treatment and its normalisation influence testing outcomes [36]. In addition, the National HIV testing campaign has increased general access to and uptake of testing, nonetheless these findings suggest that there is a need for testing and in particular a focus on recent and repeat testing [6].

The results regarding testing, stigma and behaviours associated with increased risk for HIV may have been influenced by social desirability bias.

The data about discussions about HIV are limited because we lack information about the timing, nature and parties involved in these conversations. Although the inclusion of the recent testing analysis with information about a recent discussion tries to respond to the problem of reverse causality the data is still limited. The variable is included in the analysis because despite these potential limitations it highlights the importance of converstaions around HIV and testing and points to possible avenues for further exploration and intervention research.

## Conclusion

The provision of male and youth appropriate HIV testing services is required to deliver HCT services at scale in this high prevalence, rural context. HCT provides an opportunity to emphasise prevention and to link positive people to care and treatment thereby reducing infectiousness and disease spread [39]. Effective control of the epidemic in South Africa requires massive scale up of alternate models of HCT alongside facility-based testing to provide targeted and responsive services to women, men and young people. Strong evidence exists that community-based HCT (mobile and home-based) approaches are capable of reaching a wider range of target groups with coverage in both rural and urban locations and to address the convenience factors typically associated with health facility-based HCT [40–43] in sub-Saharan Africa. These particular target groups may require even more innovative approaches to testing and access though to ensure they are reached. Targeting these and other hard to reach key populations not reflected in this research requires the provision of multiple options and a range of approaches for testing, in order to maximise the opportunities to test and engage with services. Alternative options for accessing testing that bypasses many of the acknowledged barriers may include offering testing in work and social spaces where men in particular gather. Another opportunity for testing that has the potential to create opportunities for potential disclosure in a supportive environment is couples testing [40]. Although not currently available preliminary research suggests that self-testing may present welcome opportunities for testing target groups that struggle to access facility-based testing [41, 42].

Men and younger people should also be targeted through these methods earlier and younger, school-based testing provides an opportunity to test young people easily, but there is a need to overcome political and social difficulties that affect school-based testing and has remained a challenge in the South African system to date. These should be widely implemented alongside other models of testing to facilitate uptake in South Africa [43]. The role of discussions in influencing HIV testing holds promise for its potential in shifting norms about risk perception, treatment and ultimately stigma and discrimination in this context.
